# The genetic and molecular features of the intronic pentanucleotide repeat expansion in spinocerebellar ataxia type 10

**DOI:** 10.3389/fgene.2022.936869

**Published:** 2022-09-15

**Authors:** Tatsuaki Kurosaki, Tetsuo Ashizawa

**Affiliations:** ^1^ Department of Biochemistry and Biophysics, School of Medicine and Dentistry, University of Rochester, Rochester, NY, United States; ^2^ Center for RNA Biology, University of Rochester, Rochester, NY, United States; ^3^ Stanley H. Appel Department of Neurology, Houston Methodist Research Institute and Weil Cornell Medical College at Houston Methodist Houston, TX, United States

**Keywords:** spinocerebellar ataxia type 10, intronic repeat expansion, pentanucleotide repeat, repeat interruption, RNA-gain-of-function mechanism

## Abstract

Spinocerebellar ataxia type 10 (SCA10) is characterized by progressive cerebellar neurodegeneration and, in many patients, epilepsy. This disease mainly occurs in individuals with Indigenous American or East Asian ancestry, with strong evidence supporting a founder effect. The mutation causing SCA10 is a large expansion in an ATTCT pentanucleotide repeat in intron 9 of the *ATXN10* gene. The ATTCT repeat is highly unstable, expanding to 280–4,500 repeats in affected patients compared with the 9–32 repeats in normal individuals, one of the largest repeat expansions causing neurological disorders identified to date. However, the underlying molecular basis of how this huge repeat expansion evolves and contributes to the SCA10 phenotype remains largely unknown. Recent progress in next-generation DNA sequencing technologies has established that the SCA10 repeat sequence has a highly heterogeneous structure. Here we summarize what is known about the structure and origin of SCA10 repeats, discuss the potential contribution of variant repeats to the SCA10 disease phenotype, and explore how this information can be exploited for therapeutic benefit.

## Introduction

Non-coding microsatellite repeat expansions are responsible for a wide range of dominantly and recessively inherited autosomal or X-linked human disorders (summarized in Table 1). Compared with microsatellite repeats in coding regions, non-coding microsatellite repeats tend to be more unstable, resulting in massive repeat expansions of hundreds to thousands of repeats (Table 1). However, the disease mechanisms related to these repeats in non-coding regions remain largely uncharacterized.

**TABLE 1:** Noncoding microsatellite repeat expansion diseases.

Disease	Gene	Chr	Location	Inheritance	Repeat motif	Alternative motif/interruption	Normal	Pathogenic	Adjacent retrotransposon	References
Amyotrophic lateral sclerosis/frontotemporal dementia (ALS/FTD)	*C9ORF72*	9p21.2	5′UTR/intron	AD	GGGGCC		2–24	45–2,100	N	Renton et al. (2011)
										DeJesus-Hernandez et al. (2011)
										Gijselinck et al. (2016)
Cerebellar ataxia, neuropathy and vestibular areflexia syndrome (CANVAS)	*RFC1*	4p14	intron	AR	AAGGG	AAAAG, AAAGG, AAGAG, AGAGG	11 (AAAAG)	400–2000	Y (AluSx)	Cortese et al. (2019)
Desbuquois dysplasia 2 (DBQD2)	*XYLT1*	16p12.3	promoter	AR	GGC	AGC, GGA	9–20	100–800	N	Lacroix et al. (2019)
Myotonic dystrophy type 1 (DM1)	*DMPK*	19q13.32	3′UTR	AD	CTG	CCG	5–37	50–4,000	N	Mahadevan et al. (1992)
										Turner and Hilton-jones (2010)
Myotonic dystrophy type 2 (DM2)	*CNBP*	3q21.3	intron	AD	CCTG		11–30	75–11,000	Y (AluSx)	Liquori et al. (2001)
										Turner and Hilton-jones (2010)
										Kurosaki et al. (2012)
Progressive myoclonic epilepsy type 1 (EPM1)	*CSTB*	21q22.3	promoter/5′UTR	AR	CCCCGCCCCGCG		2–3	30–75	N	Lalioti et al. (1997), Lalioti et al. (1998)
Familial adult myoclonic epilepsy type 1 (FAME1)	*SAMD12*	8q24	intron	AD	TTTCA	TTTTA	11–800 (TTTTA)	440–3,680	Y (AluSq)	Cen et al. (2018), Ishiura et al. (2018)
Familial adult myoclonic epilepsy type 2 (FAME2)	*STARD7*	2q11.2	intron	AD	TTTCA	TTTTA	9–30 (TTTTA)	661–928	N	Corbett et al. (2019)
Familial adult myoclonic epilepsy type 3 (FAME3)	*MARCH6*	5p15.2	intron	AD	TTTCA	TTTTA	9–20 (TTTTA)	791–1,035	Y (AluSx)	Florian et al. (2019)
Familial adult myoclonic epilepsy type 4 (FAME4)	*YEATS2*	3q27.1	intron	AD	TTTCA	TTTTA	4–1,219 (TTTTA)	1,000–1,600	Y (AluSx)	Cen et al. (2018), Yeetong et al. (2019)
Familial adult myoclonic epilepsy type 6 (FAME6)	*TNRC6A*	16p12.1	intron	AD	TTTCA	TTTTA	28 (TTTTA)	ND	Y (AluSx)	Cen et al. (2018), Ishiura et al. (2018)
Familial adult myoclonic epilepsy type 7 (FAME7)	*RAPGEF2*	4q32.1	intron	AD	TTTCA	TTTTA, TATTA	18	ND	N	Ishiura et al. (2018)
Fuchs endothelial corneal dystrophy type 3 (FECD3)	*TCF4*	18q21.2	intron	AD	CCCTCT		12–40	50–3,000	N	Mootha et al. (2014), Wieben et al. (2021)
Friedreich ataxia (FRDA)	*FXN*	9q21.11	intron	AR	GAA		6–27	44–1,700	Y (AluSx)	Campuzano et al. (1996), Montermini et al. (1997), Al-mahdawi et al. (2018)
Fragile X syndrome (FXS)	*FMR1*	Xq27.3	5′UTR	XLD	CGG	AGG	6–54	>200	N	Fu et al. (1991), Willemsen et al. (2013), Hagerman et al. (2017)
Fragile X-associated tremor/ataxia syndrome (FXTAS)	*FMR1*	Xq27.3	5′UTR	XLD	CGG		6–54	55–200	N	Fu et al. (1991), Willemsen et al. (2013), Hagerman et al. (2017)
Global developmental delay, progressive ataxia, and elevated glutamine (GDPAG)	*GLS*	2q32.2	5′UTR	AR	GCA		5–38	400–1,500	N	van Kuilenburg et al. (2019)
Intellectual developmental disorder, X-linked 109 (MRX109)	*AFF2*	Xq28	5′UTR	XLR	CCG		6–25	>200	N	Knight et al. (1993), Knight et al. (1994)
Neuronal intranuclear inclusion disease (NIID)	*NOTCH2NLC*	1q21.2	5″UTR	AD	CGG	AGG	9–43	90–180	N	Ishiura et al. (2019)
Oculopharyngodistal myopathy-1 (OPDM1)	*LRP12*	8q22.3	5′UTR	AD	CGG	CGT	13–45	90–130	N	Ishiura et al. (2019)
Oculopharyngodistal myopathy-2 (OPDM2)	*GIPC1*	19p13.12	5′UTR	AD	GGC		12–32	70–138	N	Deng et al. (2020), Xi et al. (2021)
Oculopharyngeal myopathy with leukoencephalopathy (OPML1)	*NUTM2B-AS1*	10q22.3	noncoding	AD	CGG	CCG	3–16	700	N	Ishiura et al. (2019)
Spinocerebellar ataxia 8 (SCA8)	*ATXN8OS* (noncoding)/*ATXN8* (coding)	13q21	noncoding/coding	AD	CTG/CAG	CTA, CTC, CCA, CTT, CCG	16–37	107–127	N	Koob et al. (1999), Moseley et al. (2000), Moseley et al. (2006)
Spinocerebellar ataxia 10 (SCA10)	*ATXN10*	22q13.31	intron	AD	ATTCT	ATTGT, ATCCC, ATCCT, ATTCC, TTTCT, ATATTCT, ATTTTCT, ATTCTCT, ATTCTTCT	9–32	280–4,500	Y (AluSx)	Matsuura et al. (2000), Kurosaki et al. (2009), McFarland et al. (2013)
Spinocerebellar ataxia 12 (SCA12)	*PPP2R2B*	5q32	5′UTR	AD	CAG		4–31	51–78	N	Holmes et al. (1999), Srivastava et al. (2017)
Spinocerebellar ataxia 31 (SCA31)	*BEAN1/TK2*	16q21	intron	AD	TGGAA	TAAAA, TAGAA, TAAAATAGAA	8–140 (TAAAA)	>500	Y (AluSx)	Sato et al. (2009)
Spinocerebellar ataxia 36 (SCA36)	*NOP56*	20p13	intron	AD	GGCCTG		3–14	650–2,500	N	Kobayashi et al. (2011), García-Murias et al. (2012)
Spinocerebellar ataxia 37 (SCA37)	*DAB1*	1p32	intron	AD	TTTCA	TTTTA	7–400 (TTTTA)	150–250	Y (AluJb)	Seixas et al. (2017)
X-linked dystonia-parkinsonism (XDP)	*TAF1*	Xq13.1	intron	XLR	CCCTCT		None	35–52	Y (SINE-VNTR-Alu)	Bragg et al. (2017)

**FIGURE 1:**
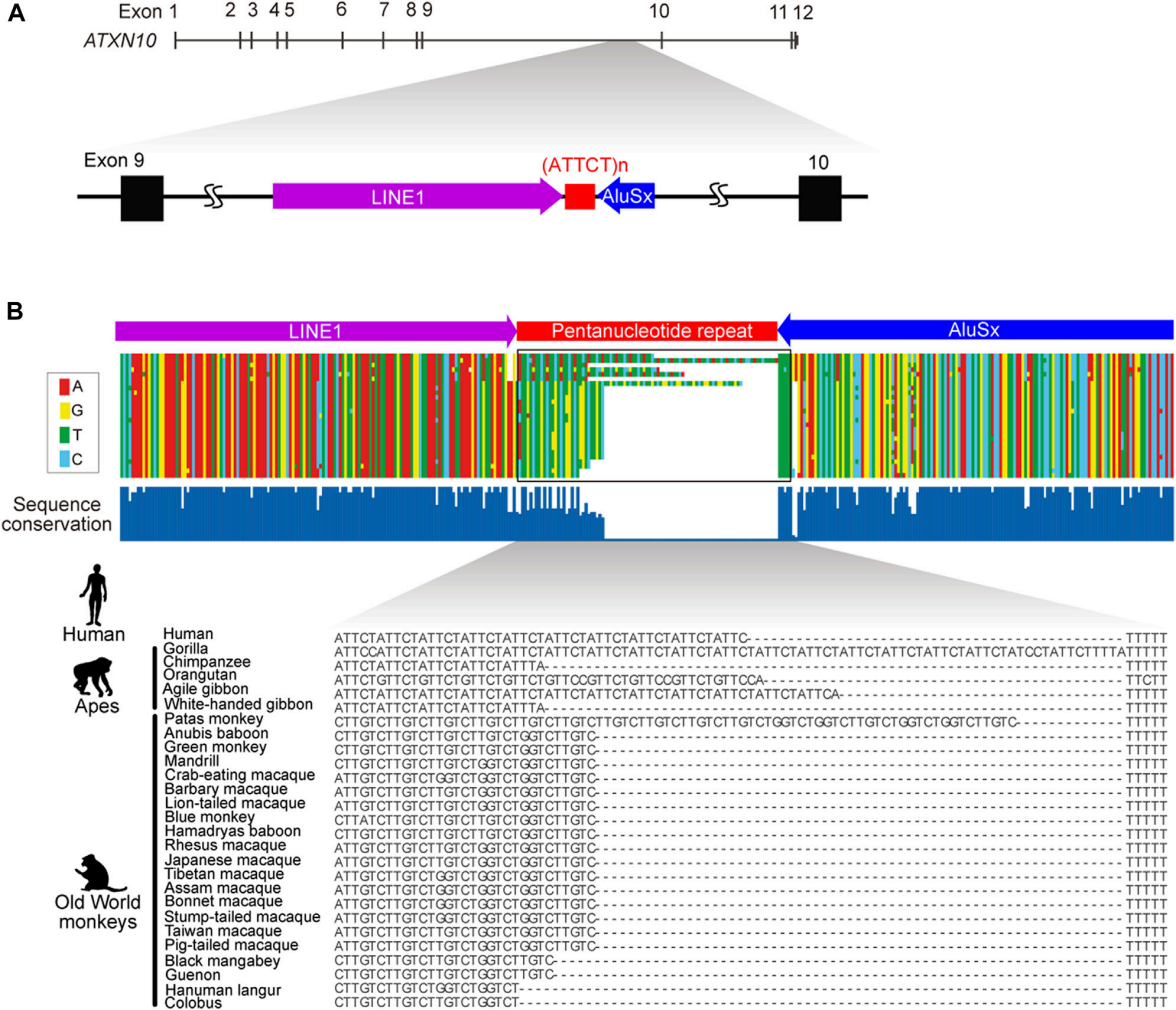
Genomic structure of *ATXN10* pentanucleotide repeats. **(A)** The genomic location of the ATTCT pentanucleotide repeat between transposable LINE1 and the *AluSx* element in human *ATXN10* intron 9. **(B)** Multiple alignments comparing *ATXN10* pentanucleotide repeats in primate species using sequence data from Kurosaki et al., 2009.

Spinocerebellar ataxia type 10 (SCA10) is an autosomal dominant neurodegenerative disorder that presents clinically with progressive cerebellar ataxia variably associated with epilepsy (Matsuura et al., 1999; Matsuura et al., 2000; Lin and Ashizawa, 2003; Lin and Ashizawa, 2005). SCA10 was the first human genetic disorder discovered to be caused by an expanded intronic pentanucleotide (ATTCT) repeat in intron 9 of the *ATXN10* gene on chromosome 22q13.3 (Figure 1) (Matsuura et al., 1999; Matsuura et al., 2000). Normal individuals usually have 9–32 ATTCT repeats, but SCA10 patients can have up to 4,500 (∼22.5 kb) repeats (Matsuura et al., 2000; McFarland et al., 2013). Since the first discovery of the SCA10 mutation, many diseases have subsequently been reported to be caused by expanded intronic pentanucleotide repeats, including SCA31, SCA37, familial adult myoclonic epilepsy (FAME), and cerebellar ataxia, neuropathy and vestibular areflexia syndrome (CANVAS). Nevertheless, the SCA10 repeat is one of the largest expansions reported to date in microsatellite repeat expansion disorders (Table 1).

The length of the expanded ATTCT repeat is highly unstable, especially during paternal transmission, and shows a variable degree of somatic and germline instability (Matsuura et al., 2004). Disease onset is usually in early adulthood, although initial symptoms can occur in teenagers and the elderly (Grewal et al., 2002; Bushara et al., 2013). While some families show conspicuous anticipation, others do not, suggesting that the genetic mechanisms underlying SCA10 are complex (Matsuura et al., 2000; Rasmussen et al., 2001; Grewal et al., 2002; Teive et al., 2004).

SCA10 can be diagnosed by southern blotting, repeat-primed PCR, or long-range PCR, which detect the repeat expansion (Matsuura et al., 2000; Matsuura et al., 2004; Matsuura et al., 2006; Kurosaki et al., 2008). However, due to technical limitations inherent in Sanger sequencing for reading long repetitive sequences (>∼1 kb), the sequence structure of SCA10 repeats was uncertain for a long time. However, recent progress in repeat-primed PCR coupled with pulse-field capillary electrophoresis or long-read sequencing technology, e.g., single-molecule real-time (SMRT) sequencing, has enabled the definition of the entire SCA10 repeat expansion sequence structure, providing new insights into the repetitive sequence and associated disease phenotypes (Mcfarland et al., 2015; Schüle et al., 2017; Hashem et al., 2020). In this review, we discuss recent progress in SCA10 research, focusing on its molecular genetics, sequence structure, and related disease mechanisms. In doing so, we explore the potential contribution of variant repeats to the SCA10 disease phenotype and explore how this information can be exploited for therapeutic benefit.

### The clinical features of SCA10

Patients with SCA10 are characterized by the core clinical phenotype of progressive cerebellar ataxia, and although epilepsy is frequently observed, its occurrence is more variable within and between families than ataxia (Matsuura et al., 1999; Zu et al., 1999; Matsuura et al., 2000; Grewal et al., 2002; Seixas et al., 2005; Raskin et al., 2007; Teive et al., 2010; de Castilhos et al., 2014; Schüle et al., 2017; Domingues et al., 2019; Domingues et al., 2019; Nascimento et al., 2019; Ramirez-Garcia et al., 2022) (Table 2). SCA10 has been reported in Mexican, Brazilian, Colombian, Argentinian, Peruvian, Bolivian, or Venezuelan families with Indigenous American ancestry (Matsuura et al., 2000; Rasmussen et al., 2001; Alonso et al., 2007; Gatto et al., 2007; Gatto et al., 2007; Raskin et al., 2007; Roxburgh et al., 2013; Leonardi et al., 2014; Baizabal-Carvallo et al., 2015; Paradisi et al., 2015; Domingues et al., 2019; Ramirez-Garcia et al., 2022) and in Chinese and Japanese families (Wang et al., 2015; Naito et al., 2017; Mao et al., 2022). A majority (∼68%) of SCA10 patients exhibit pure cerebellar ataxia but, highlighting the clinical heterogeneity, only ∼5%–7% have epilepsy in Brazilian populations from the Parana and Santa Catarina states (Domingues et al., 2019; Domingues et al., 2019) but ∼65% of patients from other regions of Brazil develop seizures (de Castilhos et al., 2014). Similarly, epilepsy frequency in Mexican families ranges anywhere from 20% to 80% (Matsuura et al., 1999; Zu et al., 1999; Rasmussen et al., 2001; Grewal et al., 2002; Teive et al., 2004; Teive et al., 2010; Schüle et al., 2017). While SCA10 is usually diagnosed in patients aged 14–48 years (Table 2), one patient from Minnesota developed SCA10 ataxia at 83 years of age (Bushara et al., 2013). Of note, SCA10 patients with epilepsy tend to be younger (24 ± 16 years) than patients without epilepsy (35 ± 9 years) (Domingues et al., 2019). In addition to cerebellar ataxia and epilepsy, patients in some families also exhibit cognitive impairment and peripheral neuropathy (Table 2). Indeed, a recent magnetic resonance imaging (MRI) study of Mexican SCA10 patients has revealed neurodegeneration not only in the cerebellum but also in other brain regions, including the brainstem, thalamus, and putamen (Hernandez-Castillo et al., 2019).

**TABLE 2:** The clinical features of SCA10.

Clinical features (%)	Domingues et al. (2019)	Nascimento et al. (2019)	de Castilhos et al. (2014)	Teive et al. (2010)	Schüle et al. (2017)	Teive et al. (2010)	Rasmussen et al. (2001)	Zu et al. (1999)	Paradisi et al. (2015)	Teive et al. (2010)	Teive et al. (2010)
Gait ataxia	99	95	100	100	80	100	94	100	100	100	100
Dysarthria	96	94	95	100	80	NA	88	100	100	NA	NA
Nystagmus	85	87	85	NA	80	NA	29	100	33	NA	NA
Dysphagia	12	8	95	100	NA	NA	NA	NA	NA	NA	NA
Dysmetria	NA	NA	NA	NA	NA	NA	88	NA	33	NA	NA
Dysdiadochokinesia	NA	NA	NA	NA	80	NA	82	NA	33	NA	NA
Slow saccades/ocular apraxia	7	6	70	NA	60	NA	71	NA	17	NA	NA
Ophthalmoplegia	11	10	NA	NA	NA	NA	NA	0	NA	NA	NA
Tremor/peripheral neuropathy	7	6	31	0	60	66	35	NA	0	NA	NA
Pyramidal signs/hyperreflexia/spasticity	1	NA	80	11	20	42	29	0	0	NA	40
Babinski sign	NA	1	NA	NA	0	NA	29	NA	NA	NA	NA
Hypotonia	NA	NA	NA	NA	0	NA	24	NA	NA	NA	NA
Hyporeflexia	NA	2	10	NA	NA	NA	24	NA	17	NA	NA
Cognitive impairment/depression	2	4	10	NA	100	NA	53	NA	17	NA	NA
Epilepsy/deizure	7	5	65	4	60	72	72	20	33	80	100

Affected individuals (n)	91	84	23	80	5	19	17	10	6	5	5
Ethnic group	Brazilian	Brazilian	Brazilian	Brazilian	Mexican	Mexican	Mexican	Mexican	Venezuelan	Venezuelan	Argentinean
Gender (male %)	48	46	NA	50	40	NA	24	NA	NA	NA	NA
Age of onset (years)	34 ± 9	35 ± 10	34 ± 10	∼36	37–48	∼27	14–44	NA	33–46	∼14	∼35
Disease duration (years)	10 ± 9	10 ± 9	13 ± 8	∼15	16–36	NA	1–34	NA	NA	NA	NA
Expansion (ATTCT)n	1842 ± 341	1874 ± 422	NA	∼1820	1,000–1,400	∼2,838	1,300–4,140	NA	NA	∼4,400	∼1,100

The most common signs and symptoms of cerebellar dysfunction in SCA10 patients are gait ataxia, dysarthria, and nystagmus (Table 2). The Scale for Assessment and Rating of Ataxia (SARA), a semi-quantitative instrument to assess impairment from ataxia, has been validated and is correlated with quality of life in SCA patients (Schmitz-Hübsch et al., 2006). The SARA score correlates positively with disease duration in SCA10 patients (r = 0.89, *p* < 0.0001) (Zonta et al., 2022). The disease progression rate, calculated as the SARA score divided by total disease duration in years, is slower in SCA10 than in other SCAs (e.g., SCA10 = 0.84; SCA2 = 1.16; SCA3 = 1.53) (Tensini et al., 2017; Zonta et al., 2022).

### Geographic distribution and origin of the SCA10 repeat expansion

SCA10 is mainly reported in individuals from Latin American countries such as Mexico (Matsuura et al., 2000; Matsuura et al., 2002; Rasmussen et al., 2001; Alonso et al., 2007), Brazil (Raskin et al., 2007; Teive et al., 2010; Domingues et al., 2019; Nascimento et al., 2019), Peru (Leonardi et al., 2014), Bolivia (Baizabal-Carvallo et al., 2015), Venezuela (Teive et al., 2010; Paradisi et al., 2015), Colombia (Roxburgh et al., 2013), or Argentina (Gatto et al., 2007; Teive et al., 2010) but not in European, African, South Asian, or Oceanic countries. The geographic distribution strongly supports a founder effect in the SCA10 allele. The subsequent identification of one SCA10 patient with Sioux Indigenous American ancestry and no Hispanic or Latino heritage solidified the hypothesis that SCA10 originates from the Indigenous American population (Bushara et al., 2013). Haplotype analyses of SCA10 patients in Latin America showed a common haplotype of six polymorphic loci, i.e., four SNPs (rs5764850-C/A, rs72556348-G/A, rs72556349-G/A, rs72556350-C/T) and two dinucleotide repeats (D22S1140 and D22S1153). Strikingly, SCA10 families typically share the common or closely related haplotype, further strengthening the evidence that the SCA10 repeat originally emerged in Indigenous Americans migrating throughout North and South America around 7,000–15,000 years ago (Almeida et al., 2009; Bushara et al., 2013; Rodríguez-labrada et al., 2020). More recently, SCA10 families have also been reported in China (Wang et al., 2015; Mao et al., 2022) and Japan (Naito et al., 2017). Haplotype analyses of these individuals have revealed that the haplotypes (rs5764850-C, rs72556348-G, rs72556349-G, rs72556350-C) common in North and South American populations are shared by Chinese and Japanese SCA10 patients (Naito et al., 2017; Mao et al., 2022), suggesting that the SCA10 mutation initially emerged at an earlier time point in Indigenous Americans before migration from East Asia to North America.

### Evolutionary origin of the *ATXN10* ATTCT repeat

In normal individuals, *ATXN10* intron 9 repeats are typically uninterrupted repetitive ATTCT units. By contrast, repeat interruptions are a common feature in the orthologous region in other higher primates (Figure 1B) (Kurosaki et al., 2009). Comparative analysis of primate genomes has shown that the pentanucleotide repeat locates at the 3′-end of the *Alu* element in humans, apes, and Old World monkeys but is entirely absent in prosimians, New World monkeys, and other primate species (Figure 1B). The pentanucleotide repeats originally arose from the poly(A) stretch of the *Alu* element in conjunction with the RNA polymerase III TTTT terminator sequence in the opposite direction of the *ATXN10* gene around ∼50 million years ago (Kurosaki et al., 2009).

The *Alu* element is a primate-specific non-coding transposable element (TE). There are approximately one million copies of the *Alu* element in the human genome, representing ∼11% of the entire genome (Lander et al., 2001). Active *Alu* elements transcribed by RNA polymerase Ⅲ are sometimes retrotransposed into the human genome to cause several human diseases by disrupting coding sequences or splicing signals (Deininger, 2011). Thus, *Alu* elements generally suffer from purifying selection to inactivate the transposing activity by rapidly shortening and accumulating mutations in the poly(A) stretch (Roy-Engel et al., 2002; Comeaux et al., 2009). This heterogeneous poly(A) stretch then becomes a source of microsatellite repeats (Roy-Engel et al., 2002; Comeaux et al., 2009).

It is conspicuous that disease-causing microsatellite repeats are frequently observed in the vicinity of *Alu* elements, e.g., the GAA motif in Friedreich ataxia (Justice et al., 2001), the CCTG motif in myotonic dystrophy type 2 (DM2) (Kurosaki et al., 2012), the TGGAA/TAAAA motif in SCA31 (Sato et al., 2009), the TTTCA/TTTTA motif in SCA37 (Seixas et al., 2017), the CCCTCT motif in X-linked dystonia-parkinsonism (XDP) (Bragg et al., 2017), the ATTTC/ATTTT repeat motif in FAME1, FAME3, FAME4, and FAME6 (Ishiura et al., 2018; Florian et al., 2019; Yeetong et al., 2019), and AAGGG/AAAAG/AAAGG/AAGAG/AGAGG motifs in CANVAS (Cortese et al., 2019) (Table 1). Abundant *Alu* elements are supposed to lead to large genomic rearrangements during DNA replication through segmental duplication or *Alu*-*Alu*-mediated recombination (Bailey et al., 2003; Hedges and Deininger, 2007). Although TE-mediated genomic instability is potentially involved in the microsatellite repeat instability in these neurological disorders, further mechanistic studies are warranted to establish the exact pathobiology.

### Heterogeneity of SCA10 pentanucleotide repeats and associations with disease phenotype

The origin of the mutant SCA10 repeat is unknown, and there are only limited data on the molecular basis of the instability of this repeat. SCA10 repeat expansions are beyond the limits of analysis by conventional Sanger sequencing. However, recent characterization of the complex pattern of ATTCT repeat sequences in normal individuals and some SCA10 families has provided new avenues for understanding the genetic basis and molecular mechanisms underlying SCA10 (Matsuura et al., 2006; Mcfarland et al., 2014; Mcfarland et al., 2015).

In normal individuals, the repeat shows length polymorphism of 9–32 tandem ATTCT units, although some large normal alleles (≥17 repeats) have TTTCT or TTTCT-ATTGT insertions in the 3′ end of the repeat (Matsuura et al., 2006). Initial studies suggested that highly interrupted intermediate alleles of 280 and 850 repeats have reduced penetrance, whereas alleles larger than this range were thought to be fully penetrant (Matsuura et al., 2006; Raskin et al., 2007). However, recent evidence indicates that SCA10 repeat expansions containing ATCCT interruptions lead to contractions during paternal transmission, with no correlation between repeat size and age of onset (McFarland et al., 2013; Mcfarland et al., 2014), contradicting the classical rule of genetic anticipation in repeat expansion diseases. Furthermore, data indicate that ATCCT interruptions appear to be a significant risk factor for an epileptic phenotype in SCA10 (McFarland et al., 2013; Mcfarland et al., 2014). By contrast, pure ATTCT expansion is sometimes associated with parkinsonism (Schüle et al., 2017). These observations suggest that the mechanisms of disease associated with the repeat structure in SCA10 are complex.

Recent progress in next-generation DNA sequencing technology, especially single-molecule real-time (SMRT) sequencing permitting exceptionally long read lengths, has made it possible to determine the entire SCA10 expansion sequence. SCA10 expansions are frequently interrupted by ATCCT ATCCC, ATTCC, ATTTCT, ATATTCT, ATTCTTCT, or ATTCTTCT (Table 1) (Mcfarland et al., 2015; Landrian et al., 2017). Given that certain types of repeat expansion, which typically consist of the TTTCA motif but not the TTTTA motif, accompany the disease phenotype in SCA37, FAME1, FAME2, FAME3, FAME4, FAME6, and FAME7 (Table 1), variant repeats in SCA10 may differentially contribute to the disease phenotype.

At the molecular level, short tandem repeats tend to change the repeat length by forming stable hairpin structures to induce misalignment of DNA strands during DNA replication, which is sometimes prone to expansion (Pearson et al., 2005). Repeat interruptions are generally thought to function as a repeat stabilizing factor by disrupting the long hairpin structure to reduce replication slippage and protect the repeats from expansion (Richards, 2001; Chintalaphani et al., 2021). Additionally, repeat interruptions modulate disease penetrance and severity, which are widely reported in several repeat expansion disorders such as DM1, SCA2, SCA8, and Huntington’s disease (Moseley et al., 2000; Sobczak and Krzyzosiak, 2005; Moseley et al., 2006; Braida et al., 2010; Wright et al., 2019). However, how these repeat interruptions drive the disease phenotype remains poorly understood.

**FIGURE 2:**
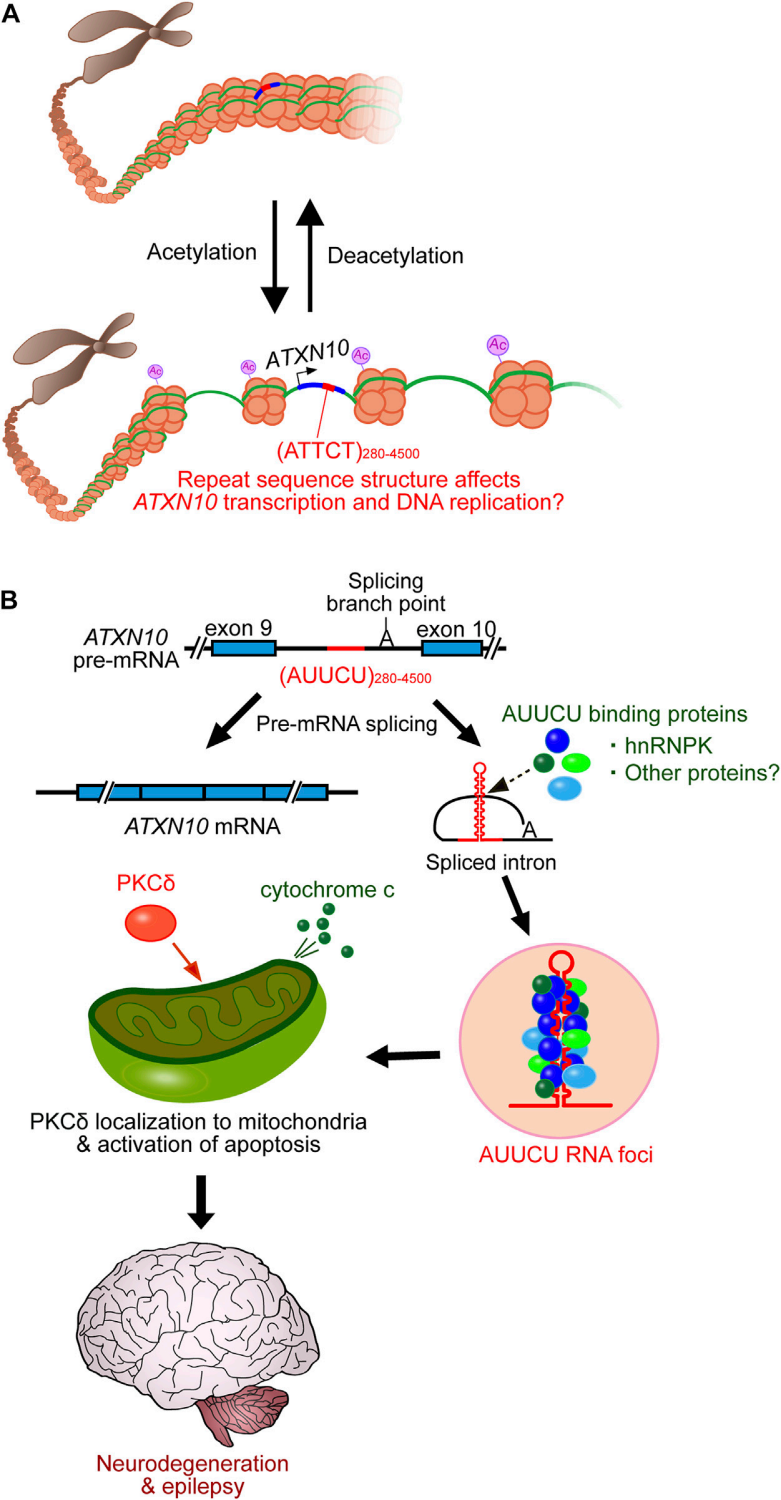
SCA10 disease model. **(A)** SCA10 ATTCT repeat expansion promotes histone acetylation, which subsequently enhances transcription and DNA replication, which may be affected by repeat interruptions. **(B)** Expanded AUUCU repeats trap hnRNPK in RNA accumulations (RNA foci). Dysfunction of hnRNPK induces PKCδ localization to mitochondria, which subsequently triggers cytochrome c release from mitochondria, activating apoptosis and eventually triggering neurodegeneration.

SCA10 repeat interruptions such as ATTTTCT and ATATTCT appear to strengthen assembly with hyperacetylated histones (Hagerman et al., 2009). Since histone acetylation is critical for chromatin disassembly and transcriptional activation (Verdin and Ott, 2015), SCA10 repeat interruptions may upregulate gene expression and induce the accumulation of expanded repeats relative to pure repeats, influencing the SCA10 disease phenotype (Figure 2A). Additionally, histone acetylation may also play a significant role in chromatin decompaction to promote DNA replication (Ruan et al., 2015). The long ATTCT repeat has been shown to function as a DNA unwinding element to induce aberrant DNA replication (Liu et al., 2007). Thus, repeat interruptions may also be involved in the DNA replication process (Figure 2A). However, further studies on the relationship between the interrupted structure of SCA10 repeats and the underlying molecular mechanisms are still needed.

### Molecular mechanisms of SCA10: Toxic RNA-mediated gain-of-function

*ATXN10* mRNAs are abundantly expressed in the juvenile (10-day-old) and adult (4-month-old) mouse (Matsuura et al., 2000) and human brain as well as the heart, skeletal muscle, and kidney (Wakamiya et al., 2006). Ataxin 10 appears to play an essential role in neurite genesis (Waragai et al., 2006) and cerebellar neuron survival (März et al., 2004). However, there is no aberrant expression of *ATXN10* (including splicing abnormalities) or of its flanking genes *FBLN1* and *PPARA* in SCA10 lymphoblasts, fibroblasts, and myoblasts (Wakamiya et al., 2006). Moreover, while *Atxn10* knockout mice show embryonic lethality, heterozygous (*Atxn10* ^+/−^) mice have no motor phenotype (Wakamiya et al., 2006). In addition, individuals harboring a translocation of chromosome 2p25.3 into intron 2 of *ATXN10* (22q13.31) show no SCA10-like symptoms, indicating that *ATXN10* haploinsufficiency does not cause the disease (Keren et al., 2010). Thus, a gain or loss of *ATXN10* function is unlikely to be the main pathogenic mechanism in SCA10.

Similar to other non-coding repeat expansion disorders, SCA10 is proposed to be caused by a toxic RNA-mediated gain-of-function mechanism (White et al., 2010; White et al., 2012). The AUUCU expansions form RNA accumulations, detected as RNA foci in the nucleus and cytoplasm of SCA10 fibroblasts and lymphoblasts (White et al., 2010). The RNA foci trap heterogeneous nuclear ribonucleoprotein K (hnRNPK) and compromise its function. This dysfunction in hnRNPK induces translocation of protein kinase C (PKC)δ to mitochondria, which subsequently induces an apoptotic pathway by releasing cytochrome c and activating caspase 3 in SCA10 cells (Figure 2B) (White et al., 2010; White et al., 2012). A transgenic mouse model of SCA10 expressing 500 ATTCT repeats within the 3′UTR of the *LacZ* gene, driven by the prion (*Prnp*) promoter (White et al., 2012), shares many phenotypic similarities with SCA10 patients, including irregular gait, increased seizure susceptibility, and neuronal loss in the cerebral cortex and hippocampus. However, this mouse does not recapitulate the cerebellar degeneration typically seen in SCA10 in humans (White et al., 2012). Thus, efforts are underway to further model the SCA10 disease phenotype, for instance, by introducing ATTCT repeats with or without repeat interruptions into the intronic region of a gene using either a pan-neuronal neuronal enolase (*Eno2*) promoter or a Purkinje cell-specific Purkinje cell protein-2 (*Pcp2*) promoter to express more expanded repeats in the cerebellum and brainstem (McFarland and Ashizawa, 2012). Furthermore, it remains unclear whether other RNA-binding proteins play a role in SCA10 pathogenesis to cause the variable SCA10 phenotype.

An alternative pathogenetic mechanism for SCA10 is bidirectional transcription producing toxic antisense transcripts or repeat-associated non-ATG (RAN) translation, as observed in DM1, DM2, fragile X-associated tremor/ataxia syndrome (FXTAS), C9orf72 amyotrophic lateral sclerosis and frontotemporal dementia ALS/FTD, and SCA8 (Zu et al., 2011; Ash et al., 2013; Todd et al., 2013; Zu et al., 2013; Zu et al., 2017). Translation of SCA10 AUUCU repeats would produce Ile-Leu-Phe-Tyr-Ser (ILFYS) pentapeptide repeats. However, there has yet to be a study of these pentapeptides in SCA10 cells and SCA10 mice, and a thorough molecular analysis is still required.

### Moving towards effective SCA10 therapeutics

The molecular basis of the unstable repeat expansion and the underlying disease mechanism in SCA10 patients are still poorly characterized, hampering efforts to develop effective therapeutics for affected individuals. The AUUCU repeat forms an unusual hairpin structure *in vitro* via the hydrogen bonds formed between A-U and U-U base pairs (Handa et al., 2005). The small molecule dimeric compound 2AU-2 selectively binds to A-U base pairs to disrupt RNA folding and AUUCU accumulations in SCA10 fibroblasts (Yang et al., 2016). Furthermore, 2AU-2 treatment effectively reduced PKCδ localization to mitochondria and reduced apoptosis (Yang et al., 2016). Remarkably, 2AU-2 treatment neither reduced the abundance of normal *ATXN10* transcripts nor triggered apoptosis in healthy fibroblasts (Yang et al., 2016). Thus, the bioactivity of 2AU-2, which is applicable not only to SCA10 patients but also to other AU-rich repeat expansion disorders, needs further evaluation in relevant neuronal models.

In addition to small molecules, other RNA silencing therapies could be applicable to SCA10. RNA silencing therapies include antisense oligonucleotides (ASOs) (Mceachin et al., 2020; Schwartz et al., 2021), artificial microRNAs (miRNAs) (Martier et al., 2019), CRISPR-Cas9 system approaches (Batra et al., 2017; Zhang et al., 2020), and DNAzymes (Zhang et al., 2021). SCA10 is suited to these therapeutic strategies because 1) the repeat is in the intron of both normal and expanded alleles and is not translated, so the repeat can be silenced whilst minimizing adverse consequences; 2) the silencing of this expanded pentanucleotide repeat would suppress all downstream pathogenic pathways; 3) no opposite strand transcripts containing the repeat have been detected at the SCA10 locus; and 4) preferential targeting of the mutant RNA may be feasible with therapeutics that directly engage the repeat units because of the unique and large size of mutant repeat alleles. Further mechanistic studies are likely to facilitate the development of effective therapeutics for SCA10.

## Conclusion

SCA10 was the first human genetic disorder discovered to be caused by an expanded intronic pentanucleotide repeat. Since the first discovery of the *SCA10* mutation, many diseases have subsequently been reported to be caused by expanded intronic pentanucleotide repeats, including SCA31, SCA37, FAME, and CANVAS. Therefore, elucidating the molecular basis of SCA10 will provide more generalizable insights into the disease mechanisms underpinning similar intronic repeat expansion disorders. Furthermore, in the absence of reliable biomarkers and the variable disease onset, this molecular knowledge should provide new avenues for the development of biomarkers so that affected individuals can receive early interventions and support.

While there is currently no therapy for SCA10, advances in sequencing technology, disease models, our understanding of the genetic, transcriptomic, and proteomic features of the consequences of expanded SCA10 repeats, and developments in nucleic acid-based therapies are likely to contribute to the development of a clinically translatable strategy to detect and treat patients with SCA10 and other similar neuronal disorders.
